# Bumblebee Venom Serine Protease Increases Fungal Insecticidal Virulence by Inducing Insect Melanization

**DOI:** 10.1371/journal.pone.0062555

**Published:** 2013-04-23

**Authors:** Jae Su Kim, Jae Young Choi, Joo Hyun Lee, Jong Bin Park, Zhenli Fu, Qin Liu, Xueying Tao, Byung Rae Jin, Margaret Skinner, Bruce L. Parker, Yeon Ho Je

**Affiliations:** 1 Department of Agricultural Biology, College of Agricultural & Life Sciences, Chonbuk National University, Jeonju, Korea; 2 Department of Agricultural Biotechnology, College of Agriculture & Life Sciences, Seoul National University, Seoul, Korea; 3 Department of Applied Biology, College of Natural Resources and Life Science, Dong-A University, Busan, Korea; 4 Entomology Research Laboratory, University of Vermont, Burlington, Vermont, United States of America; 5 Research Institute for Agriculture and Life Sciences, Seoul National University, Seoul, Korea; Cinvestav, Mexico

## Abstract

Insect-killing (entomopathogenic) fungi have high potential for controlling agriculturally harmful pests. However, their pathogenicity is slow, and this is one reason for their poor acceptance as a fungal insecticide. The expression of bumblebee, *Bombus ignitus,* venom serine protease (VSP) by *Beauveria bassiana* (ERL1170) induced melanization of yellow spotted longicorn beetles (*Psacothea hilaris*) as an over-reactive immune response, and caused substantially earlier mortality in beet armyworm (*Spodopetra exigua*) larvae when compared to the wild type. No fungal outgrowth or sporulation was observed on the melanized insects, thus suggesting a self-restriction of the dispersal of the genetically modified fungus in the environment. The research is the first use of a multi-functional bumblebee VSP to significantly increase the speed of fungal pathogenicity, while minimizing the dispersal of the fungal transformant in the environment.

## Introduction

Insect killing (entomopathogenic) fungi have high potential in controlling agriculturally harmful pests [1]. Some products have been industrialized as follows: *Beauveria bassiana* (e.g., BotaniGard® (BioWorks), Mycotrol® (Koppert), and Boverin® (Biodron)), *Beauveria brongniartii* (Betel® (Natural Plant Protection)), *Lecanicillium longisporum* (Vertalec® (Koopert)), *Metarhizium acridum* (Green Muscle® (CABI Bioscience)), *Metarhizium flavoviride* (Biogreen® (Becker Underwood)), and *Isaria fumosorosea* (PreFeRal® (Biobest) and Priority® (T. Stanes & Company)) [2]. The main active components of these commercial products are conidia (asexual spores) with high variability in virulence and slow pathogenesis [3], thus having difficulties in the expansion of fungal insecticide market [4].

So far, some efforts have been given to the expression of pathogenesis-related genes, such as *B. bassiana* chitinase gene [5] and *Bacillus thuringiensis* vegetative insecticidal protein (VIP) gene [6] in *B. bassiana* and insect-specific scorpion neurotoxin (AaIT) gene [7] in *M. anisopliae* to increase fungal virulence. These proteins were previously reported expressed in baculovirus expression vector system (BEVS) with the assessment of insecticidal potentials. Additionally, insect cuticle-degrading fungal own Pr1 protease and fusion protein of Pr1 and chitinase gene were over-expressed in *M. anisopliae* and *B. bassiana*, respectively [8], [9]. But much more virulent entomopathogenic fungi need to be developed for efficacious pest management.

Melanization was studied as a rapid insect response to challenges of its immune system and as a novel strategy to accelerate host mortality. When arthropods encounter an immune challenge, they initiate a serine protease cascade that, in turn, leads to the activation of prophenoloxidase (proPO)-activating factors (PPAFs) [10]. These factors are activated by cleavage between clip of PPAFs and serine protease domains. Once activated, PPAFs catalyze the conversion of proPO to phenoloxidase (PO). This causes the conversion of phenols to diphenol, quinine and, finally, melanin.

Recently we found that bumblebee (*Bombus ignitus*) venom serine protease (Bi-VSP) has an arthropod PPAF function and fibrinolytic activity [11]. In some insects, Bi-VSP triggers the phenoloxidase (PO) cascade by the activation of PPAF. Injection of purified Bi-VSP induces a lethal melanization response in target insects by modulating the innate immune response. In mammals, Bi-VSP acts similarly to snake venom serine protease [12], which exhibits mammalian fibrinolytic activity. The fibrinolytic activity of Bi-VSP, possibly inhibiting blood coagulation, can facilitate the spread of toxic components throughout the bloodstream. Blood coagulation disorders are a global and frequently lethal medical disease. When clots are not dissolved, they accumulate in blood vessels and cause thrombosis leading to myocardial infraction and other cardiovascular diseases [13].

It is hypothesized that the fungi-based expression of Bi-VSP induces fast melanization of whole insect bodies, and the transformants possibly have much higher insecticidal potency than the previous achievements. In this work, we integrated multi-functional Bi-VSP to the insect killing fungus, *B. bassiana* ERL1170 by restriction enzyme-mediated integration method, which was confirmed by RT-PCR and western blotting, followed by a fibrinolysis assay. For the extracellular secretion of Bi-VSP protein, the active domain of the *vsp* gene was tailed to the signal sequence of *B. bassiana* chitinase. A selected transformant was injected to yellow spotted longicorn beetle larvae to confirm insect melanization and sprayed on beet armyworms to examine mortality. Our work is the first fungus-based expression of Bi-VSP, which was not available in an insect cell-mediated BEVS.

## Results

### Integration of *vsp* Gene into a Transformation Vector

**Figure 1:**
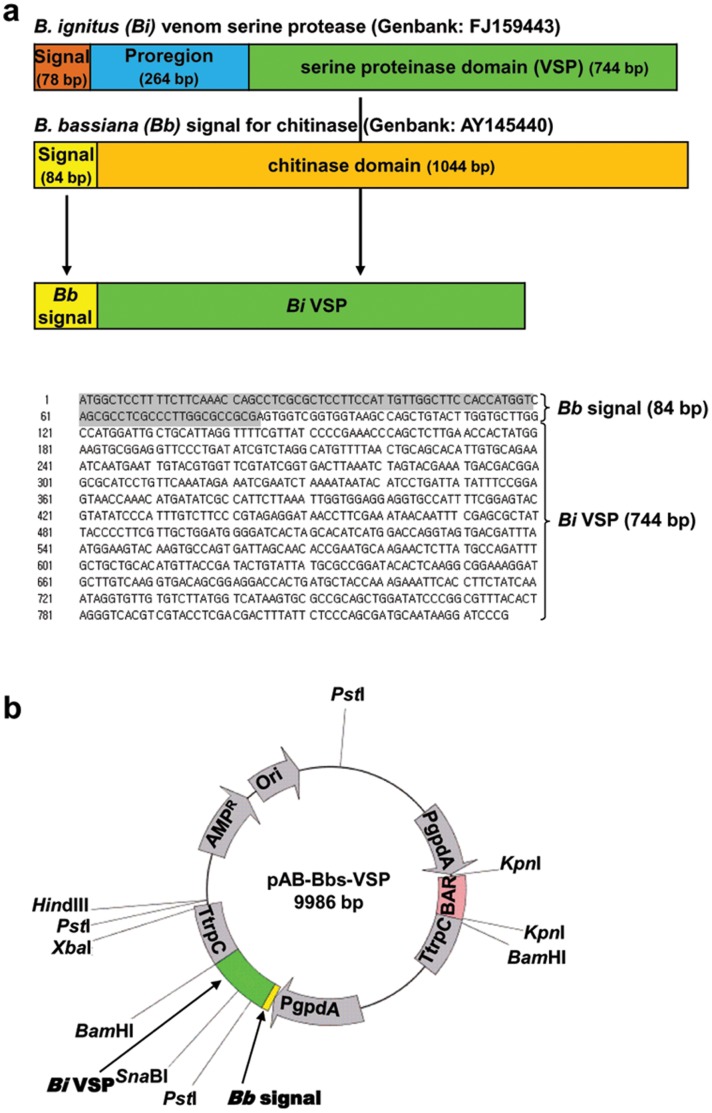
Tailing of *B. ignitus* (*Bi*) VSP fragment with *B. bassiana* (*Bb*) chitinase signal for extracellular secretion and integration of the fusion form into a fungal transformation vector. (a) A schematic diagram of the fusion of *Bb* chitinase signal and *Bi* VSP fragments. The 5′-end of *Bi* serine protease domain was tailed by 3′-end of *Bb* signal by four-round serial PCR. The fusion form of PCR product was confirmed by sequencing. *Bb* signal, shadowed; and *Bi* VSP, not-shadowed. (b) A map of fungal transformation vector, pAB-BbsVSP including the fusion form of *Bb* signal and *Bi* VSP fragment. The plasmid retains the *BAR* and *AMP* resistance genes of the parent plasmid pABeG (*BAR* is a selectable marker providing resistance to glufosinate).

For integration of the *vsp* gene into *B. bassiana* ERL1170 and the extracellular secretion of VSP protein, the active domain of the *vsp* gene was tailed with *B. bassiana* signal (*Bbs*) sequence for chitinase (**Figure 1a**) and inserted into a fungal transformation vector, yielding the binary plasmid pAB-Bbs-VSP (9.9 kb) (**Figure 1b**). A shuttle vector, pBluscript II KS(+)-egfp cassette (expression fragment) was used to insert the *Bbs-vsp* PCR products to the fungal transformation vector, pABeG provided by Dr. Feng Ming-Guang in Zhezhang University (**Figure S1**). The reason of the use of shuttle vector, pBluscript II KS(+) was that pABeG had the same promoters and terminators for *bar* and *egfp* genes, thus having difficulties in cloning. The pABeG has phosphinothricin (PPT) resistant *bar* and *egfp* genes, and each gene is expressed under the control of the same *gpdA* promoter in the same transcriptional direction. The binary plasmid, linearized by cutting with *Hin*dIII, was transformed into ERL1170 by the restriction enzyme-mediated integration based on blastospores [15].

### Expression of Bi-VSP in *B. bassiana*

**Figure 2:**
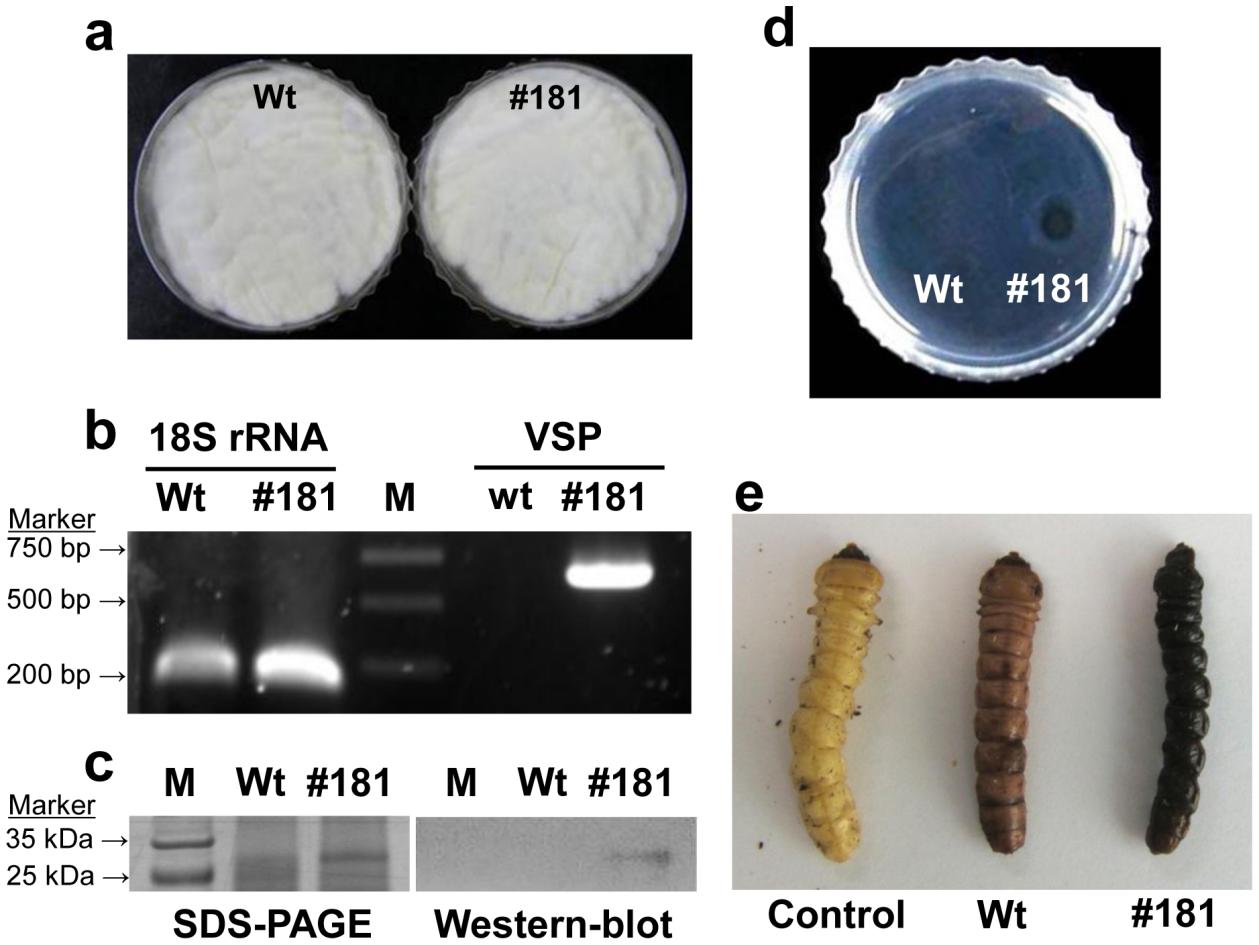
Expression of bumble bee venom serine protease (VSP) in an insect-killing fungus, *B. bassiana* ERL1170 (transformant: BbsVSP-#181). (a) Solid culture of wild type (Wt) and BbsVSP-#181 on fourth-strength Sabouraud dextrose agar (SDA/4) for 7 days. (b) RT-PCR analysis of VSP in BbsVSP-#181 (#181). M, Marker. (c) Western blot analysis of liquid-cultured BbsVSP-#181 supernatant using an antiserum to bumble bee VSP. (d) Fibrinolytic activity of BbsVSP-#181 supernatant. Serine proteases are known to have fibrinolytic activity. (e) Melanization activity of BbsVSP-#181 spores (conidia) against yellow spotted longicorn beetles 4 days post injection. Beetles were injected with conidia at 40 µl (1×10^7^ conidia ml^−1^) per larva. Phosphate buffered saline (PBS) solution was used as a base for all the treatments.

Transformation of the competent blastospores of wild type *B. bassiana* with the *Bbs-vsp*-vectoring binary plasmid produced 422 colonies on plates of Czapek’s solution agar medium containing 600 µg ml^−1^ PPT. After three rounds of subculturing on PPT-free fourth-strength Sabouraud dextrose agar (SDA/4) plates, one of the putative transformants, BbsVSP-#181 was selected. BbsVSP-#181 grew similarly to the wild type on SDA/4 plates (**Figure 2a**). White mycelial growth and in 7 days a similar number of conidial production (Wt: 1.9×10^8^±3.1×10^7^ and BbsVSP-#181∶1.8×10^8^±2.4×10^7^ conidia per cm^2^) was observed. From an economic standpoint, additional efforts are not necessary to increase BbsVSP-#181 conidial production to the level of the wild type. The transformant was consistently found to express the *Bbs-vsp* gene as determined by RT-PCR analysis (**Figure 2b**). In the Western blot, ∼27 kDa (744 bp) VSP was detected in the supernatant concentrates by its polyclonal antibody, but not from the supernatant of the wild-type strain (**Figure 2c**). Transcription of *Bbs-vsp* gene and translation followed by extracellular secretion of VSP was confirmed. Secreted VSP is possibly ready to induce PPAFs in insects after the hyphal penetration, resulting in faster pathogenesis.

To determine the biological activity of the transformant (BbsVSP-#181), a fibrin plate assay and a bioassay against yellow spotted longicorn beetle larvae were conducted. In fibrin plate assay in a 60-mm dish, supernatant concentrate (25-fold) of BbsVSP-#181 strain made a clear area (degradation of fibrin) on the fibrin plate but no corresponding clearance with the wild type (**Figure 2d**). BbsVSP-#181 strain had supernatant dosage-dependent fibrinogen degradation activity when fibrinogen clots were submerged to the supernatant (**Figure S2**). Given the previous report describing fibrinolytic activity of VSP, secreted VSP from the BbsVSP-#181 strain proved to maintain its own biological functions.

When fungal conidia (40 µl of 1×10^7^ conidia ml^−1^) were injected to second instars of yellow spotted longicorn beetle larvae, BbsVSP-#181-injected larvae completely turned black in 4 days, compared to the light pink color of the wild type-injected larvae in which fungal growth proceeded slowly (**Figure 2e**). In the BbsVSP-#181 treatment, small dark brown spots were observed 2 days post-injection, followed by complete insect melanization without fungal outgrowth in 7 days, but the wild type-injected larvae turned light pink as the mycosis developed (without forming dark spots on the host cuticle) until the hosts were covered by a sporulating mass of mycelium (**Figure S3**). Consequently, production of VSP and its use as an activator for PPAFs were confirmed.

### Virulence of BbsVSP-#181 against Beet Armyworms

**Figure 3:**
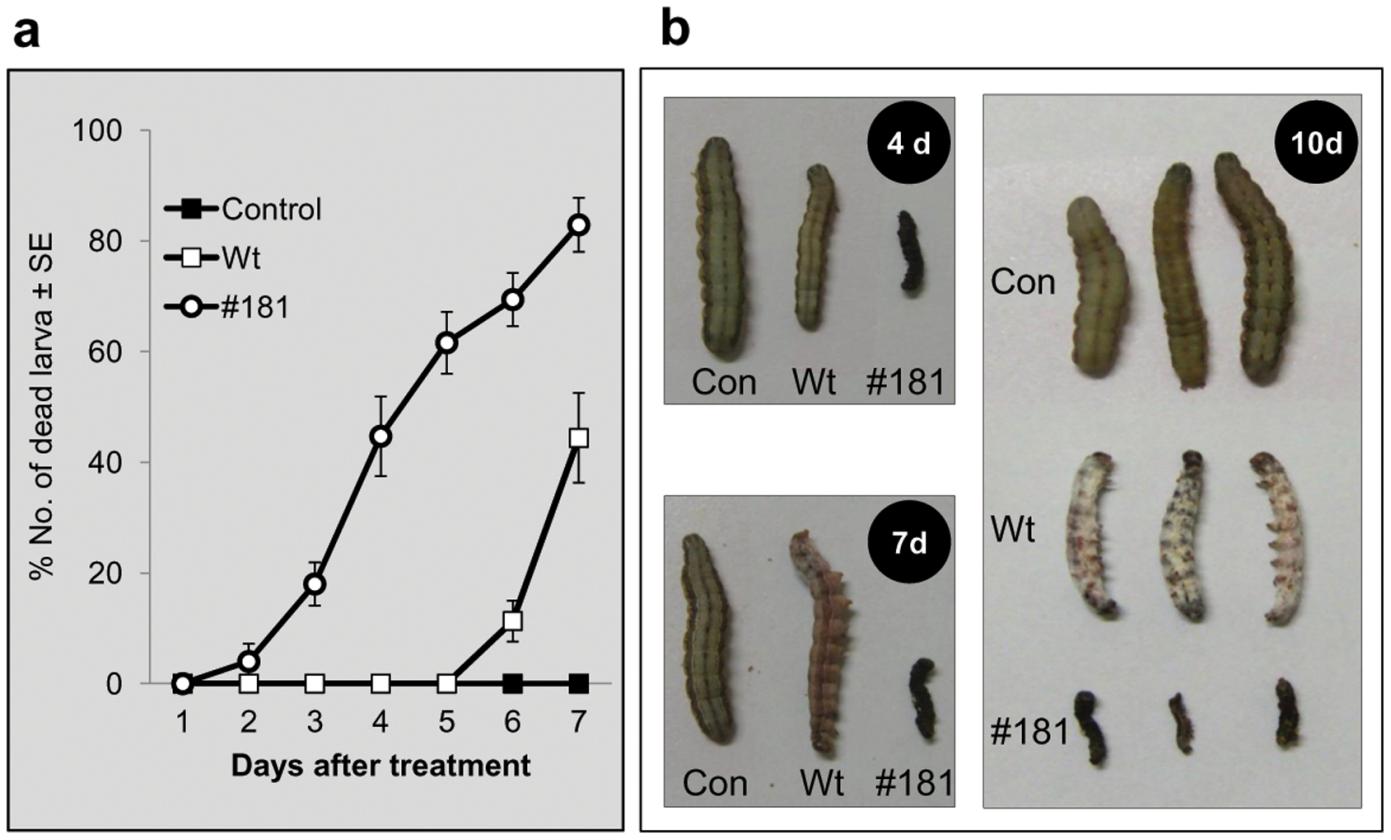
Insecticidal activity of wild type (Wt) and transformant BbsVSP-#181 (#181) against beet armyworm larvae in laboratory conditions. (a) Percentage (%) of dead beet armyworm larvae after the spray of Wt and BbsVSP-#181 spores at 1×10^7^ conidia ml^−1^ (*N* = 27). Siloxane solution (0.03%) as a surfactant was used as a base for all the treatments. (b) Symptoms of beet armyworms in 4, 7 and 10 days after the treatment. BbsVSP-#181-treated beet armyworms turned black in 4 days and no stage development was observed. But beet armyworms in the wild type treatment developed to fourth instars with mycosis in 7 days and completely mycotized in 10 days.

A transformant, BbsVSP-#181 conidial suspension (1×10^7^ conidia ml^−1^) was sprayed on 2^nd^ instar of beet armyworm (*Spodoptera exigua*) larvae under laboratory conditions to assess its pest control activity. The transformant had significantly faster virulence than the wild type in controlling beet armyworms (**Figure 3a**). To achieve 50% mortality against beet armyworms at 1×10^7^ conidia ml^−1^ dose, BbsVSP-#181 required 4.2 (±0.8) days, compared to more than 7 days for the wild type (estimated by 9.3 (±2.5) days) (**Table S1**). Thus, it takes 2.2-fold shorter time for controlling beet armyworms. BbsVSP-#181-treated beet armyworms turned black in 4 days and no further development was observed (**Figure 3b**). The melanization in beet armyworms began during the early stages of fungal pathogenesis, and soon after fungal germination and hyphal penetration. However, beet armyworms in the wild type treatment developed to 4th instar larvae with mycosis in 7 days. The wild type fungus had more time to achieve mechanical penetration and enzymatic degradation for complete mortality. Non-treated control beet armyworms developed to 5th instar in 10 days. Secondly, 7 days after the spray treatments (1×10^5^, 1×10^6^, 1×10^7^, and 1×10^8^ conidia ml^−1^), LC_50_ (lethal concentration causing 50% mortality) of BbsVSP-#181 was 3.6 (±0.8)×10^5^ conidia ml^−1^, which was significantly lower than that of wild type (41.3 (±17.1)×10^5^ conidia ml^−1^).

## Discussion

The fungus-based expression of bumblebee serine protease significantly increased the virulence of wide type, which may be compared to the expression of scorpion neurotoxin (AaIT) [7] and the over-expression of fungal own Pr1 protease [8], [9] as mentioned above. In the BbsVSP-#181 treatment, it took 2.2-fold shorter time for controlling beet armyworms. In the expression of neurotoxin, AaIT59 transformant required 4.5 days (wild type: 6.3 days) to achieve 50% mortality against Tobacco cutworms and 6.1 days (wild type: 9.9 days) against yellow fever mosquitoes. Approximately it took 1.4 to 1.6-fold shorter time for controlling the cutworms and mosquitoes. Similarly, in the over-expression of Pr1 protease, transformants required 93–96 h (wild type: 128 h) against gypsy moths and 98–121 h (wild type: 131 h) against green peach aphids. Approximately it took 1.1 to 1.4-fold shorter time for controlling the moths and aphids.

VSP-integrated insect-killing fungi have some advantages in pest management. They control agriculturally harmful insects in a short time compared to the wild type. Activation of melanization cascade is very sensitive to initiators and proceeds very quickly [17]. Thus, low levels of hyphal penetration may be enough to induce the melanization cascade when VSP-integrated fungus is applied. From an economic standpoint, VSP-integrated fungi do not need to spend great deal of energy for hyphal penetration into the haemocoel. However, in research on the expression of chitinase, vegetative insecticidal protein and insect-specific scorpion toxin (expressed in haemocoel), hyphal growth and penetration should be fully accomplished for the expression of integrated genes. Fungal penetration and expression of VSP is a strategically well combined tactic to achieve fast control with high biological performance. Particularly, VSP-integrated fungi can be more useful in controlling pests with a short-term life cycle.

Another merit of VSP-integrated insect-killing fungi is the self-restriction of further reproduction and dispersal in the environments by the genetically modified fungus. Because VSP-mediated insect melanization quickly kills target insects and the fungal pathogen that introduced this toxin, no further development by the fungus should be possible. The rapid killing of its host insects and the death of treated fungus explains why no mycoses were found in the VSP-integrated fungus treatments. Melanization improves the effectiveness of other immune responses that promote arthropod resistance to microbial infection [18] and suppresses the infection of parasitoids [19]. Dispersal of genetically modified fungi can be naturally inhibited in the environment so that the registration process may be minimized although fundamental safety tests are required. But, genetically modified fungi, even those whose reproduction or dispersal is self-limited, may or may not be easy or even possible to register in many countries merely because they are GMOs, regardless of their beneficial properties. It may take more times in the industrialization of this VSP-integrated insect-killing fungus than expectation. However, in other cases (expression of other functional insecticidal proteins such as chitinase, vegetative insecticidal protein, and insect-specific scorpion toxin), dispersal of genetically modified fungi may be usual events, so it should be carefully controlled in the environment.

VSP-integrated insect-killing fungi inherit any fungal own host spectrum, by which VSP can be expressed in potentially many insects. Among microbial pest control agents, *Bacillus thuringiensis* (Bt) and baculoviruses can be alternatively considered, but host spectrums are mainly limited to lepidopteran pests (moths) and BEVS is not available in VSP expression as described above. Insect-killing fungi, particularly *B. bassiana* virulent to many agricultural pests such as moths, aphids, mites, stink bugs, whiteflies, thrips and soil-dwelling beetles.

Mass production of entomopathogenic fungi has been effectively developed and is cost-effective [20]. Thanks to the development of industrialization technology, VSP-integrated fungal spores (conidia) or VSP proteins can be easily mass-produced and harvested. A great deal of effort has been given to increase the shelf life of fungal spores during distribution and after application. Some studies are necessary to investigate whether any directed genetic modification of a particular fungal strain does or does not affect such critically important properties of the fungus.

In conclusion, for the first time bumble bee venom serine protease (Bi-VSP) has been successfully expressed in the insect-killing fungus, *B. bassiana* ERL1170 and has caused the melanization and rapid death of yellow spotted longicorn beetle larvae and beet armyworm larvae as well as supernatant-mediated mammalian fibrinolysis. This research highlights the expression of multi-functional Bi-VSP (not available in BEVS) in a fungal platform that is especially relevant for agricultural (fungal application) and pharmacological (purified proteases) fields with much stronger biological activities. These results could significantly increase the economic value of entomopathogenic fungi for at least some specific application.

## Materials and Methods

### Microbial Strains

The wild type strain *B. bassiana* ERL1170 (ARSEF2060 in USDA-ARS in Itheca) [14] was provided by Entomology Research Laboratory, University of Vermont, USA, and maintained on fourth-strength Sabouraud dextrose agar (SDA/4) in darkness at 25°C for colony growth. *Escherichia coli* TOP10 (Invitrogen, Carlsbad, CA), used for DNA manipulation, were cultured in Luria-Bertani (LB) medium containing 50 g ml^−1^ ampicillin [15].

### Vector Construction

A fungal transformation vector, pABeG expressing *egfp* gene (provided by Dr. Feng Ming-Guang in Zhezhang University, China) was used as a plasmid backbone and its availability in *B. bassiana* was confirmed. The pABeG has phosphinothricin (PPT) resistant *bar* and *egfp* genes, and each gene is expressed under the control of *gpdA* promoter in the same transcriptional direction. Active domain of *vsp* gene was tailed with *B. bassiana* signal (*Bbs*) sequence and inserted into a fungal transformation vector, yielding the binary plasmid pAB-Bbs-VSP (9.9 kb). The full-length, 744 bp active domain *vsp* gene (GenBank FJ159443) was amplified by PCR of pGEM-Bi-VSP donated by Dr. Byung Rae Jin in Dong-A University, S. Korea. For extracellular secretion [5], the 5′-end of *Bi* serine protease active domain was tailed by 3′-end of 84 bp *Bb* signal fragment for chitinase (GenBank AY145440) by four-round serial PCR (**Table S2**), finally flanked with *Bam*HI at 3′-end (*Bbs-vsp*).

A PCR product of *Bbs-vsp* was integrated into the fungal transformation vector, pABeG containing *egfp* expression cassette by exchanging *egfp* gene with *Bbs-vsp* gene with the help of the shuttle vector, pBluscript II KS(+). The 3,668 bp *egfp* expression cassette including *gpdA* promoter (P*gpdA*) and *trpC* terminator (T*trpC*) was cut from pABeG using *Bgl*II and *Hin*dIII and integrated into pBluscript II KS(+), which was previously cut using *Bam*HI (compatible end to *Bgl*II) and *Hin*dIII. The ligated plasmid was designated as pBluscript II KS(+)-egfp cassette. To integrate *Bbs-vsp* into the position of *egfp* in pBluscript II KS(+)-egfp cassette, the *Bbs-vsp* PCR product was cut using *Bam*HI and pBluscript II KS(+)-egfp cassette was cut using *Nco*I/blunted and *Bam*HI to remove *egfp* region. The insert and the vector was ligated and designated as pBluscript II KS(+)-Bbs-vsp cassette. Lastly, to integrate the *Bbs-vsp* cassette from pBluscript II KS(+)-Bbs-vsp cassette into the fungal transformation vector, pABeG, pBluscript II KS(+)-Bbs-vsp cassette was cut using *Spe*I and *Hin*dIII and pABeG was cut using *Xba*I (compatible end to *Spe*I) and *Hin*dIII, finally yielding the binary plasmid pAB-Bbs-VSP (9.9 kb) (**Figure S1**).

### Fungal Transformation

The binary plasmid, linearized by cutting with *Hin*dIII, was transformed into *B. bassiana* ERL1170 by the restriction enzyme-mediated integration based on blastospores [16]. Transformants were grown on Czapek’s solution agar containing 600 µg ml^−1^ PPT. Putative transformants were sub-cultured three times on PPT-free SDA/4 at 25°C. Genomic DNAs were extracted from 5-day old fungal mycelial mass by the quick fungal genomic DNA extraction method [21] and the presence of *bar* and *Bbs-vsp* was examined by PCR with primers Bar-F and Bar-R (5′-AGTCGACCGTGTACGTCTCC-3′ and 5′GAAGTCCAGCTGCCAGAAAC-3′) and primers Bbs-vsp-F and Bbs-vsp-R (5′- ATGGCTCCTTTTCTTCA-3′ and 5′-TCCGCTGTCACCTTGAC-3′).

### Verification of Expression

Transcription of *Bbs-vsp* in the transformants was examined by the extraction of RNAs from 5-day old fungal mycelial mass, produced in SDA/4 in darkness at 25°C, using TRIZOL (Invitrogen) method and reverse transcription PCR (RT-PCR) with the primers Bbs-vsp-F and Bbs-vsp-R. For western blotting, transformants and wild type were cultured in fourth strength Sabouraud dextrose broth (SDB/4) at 25°C and 150 rpm of shaking for 5 days. Cultured broth was filtered using 3M filter papers and syringe filters (0.25 µm) and concentrated by ultrafiltration using Amicon tubes (Millipores, MA, USA). The concentrates were subjected to 12% SDS-polyacrylamide gel electrophoresis (PAGE) and electrophoretically transferred to a polyvinylidene difluoride (PVDF) membrane. A polyclonal antibody against VSP expressed in Sf-9 insect cells through baculovirus expression vector system (BEVS), provided by Dr. Byung Rae Jin, was derived from mouse and used to detect the expression of VSP secreted from mycelia of transformants. The PVDF membrane was incubated with a 1,000-fold dilution of the polyclonal antibody and a 2,000-fold dilution of goat anti-rabbit IgG horseradish peroxidase (HRP) as the second antibody. Visualization was performed using the luminol reagent SC-2048 (Santa Cruz Biotechnology Co.).

### Fibrinolytic Activity Assay

The original fibrin plate assay [22] was slightly modified for use here in measuring the fibrinolytic activity of the supernatants. An aliquot of 5 ml fibrinogen (Sigma-Aldrich, from human plasma) (0.25%) solution in PBS (phosphate buffered saline, pH 7.4) was mixed with 10 units of thrombin (Sigma-Aldrich, from human plasma) (1 unit/50 µl) in a 60-mm Petri dish and incubated at 37°C for 15 min to speed up the clotting. A supernatant concentrate (10 µl) was dropped onto the fibrin plate. The plates were then incubated at 37°C for 2 h and visually inspected for liquefaction.

Secondly, to investigate the degradation of fibrin in supernatant solution, an aliquot of 100 µl fibrinogen (0.25%, in PBS) solution was mixed with 10 units of thrombin (1 unit/50 µl) in an 1.5-ml Eppendorf tube and incubated at 37°C for 15 min for clotting. Supernatant was loaded at 200, 400, and 800 µl tube^−1^ and the tubes were incubated at 37°C for 3 hr. After the incubation, solutions in the tubes were completely removed using a pipette and the amount of remaining fibrin was observed.

### Bioassay

Yellow spotted longicorn beetle (*Psacothea hilaris*) for fungal injection and beet armyworm (*Spodoptera exigua*) for fungal spray were supplied by the Department of Agricultural Biology, National Academy of Agricultural Science, Republic of Korea. They were reared as previously described [23], [24] and subjected to bioassays [25]. To produce test fungal spores (conidia), a transformant and wild type fungi were inoculated on SDA/4 at 100 µl (1×10^7^ conidia ml^−1^) per 60-mm diam Petri-dish and incubated in darkness at 25°C for 10 days. As inocula for injection, conidial suspensions, where hyphae were removed, were adjusted to 1×10^7^ conidia ml^−1^ using PBS for injection and 0.03% (v/v) siloxane solution (Silwet L-77) as a wetting agent. PBS and siloxane solution served as controls.

For injection, second instars of yellow longicorn beetle larvae were placed at 4°C for 20 min. Second instars of yellow longicorn beetle larvae were injected with 40 µl of hypha-free conidial suspensions filtered using cheese clothes, where a sterile needle was promptly pierced under the epidermis of soft membranous cuticle between the sixth and seventh abdominal segments. Injected larvae were placed in 60-mm diam. Petri dishes that contained artificial diets (mulberry leaf and branch powder 100 g, carrageenan 5 g, distilled water 300 ml) (1×1×0.5 cm^3^ piece per dish). The dishes were covered with lids and held in an incubator at 25±1°C and 16∶8 (L/D). Petri dishes were not stacked to keep from excess moisture from forming inside of the dishes. Symptom of melanization and mycosis was observed daily for 10 days.

In spray test, a group of 10 larvae was placed in a 60-mm Petri dish (3 dishes/treatment), and all dishes were covered with lids and held at 4°C for 20 min to reduce mobility. Fungal suspensions were sprayed at 10 ml per dish using a microsprayer, and dishes were covered with lids and sealed with Parafilm. They were held in an incubator at 25±1°C and 16∶8 (L/D). Petri dishes were not stacked to keep from excess moisture from forming inside of the dishes. Mortality was assessed by counting the number of live and dead larvae per dish daily for 7 days. This entire bioassay was repeated twice using different batches of conidial suspensions on different days. Secondly, to determine lethal concentration causing 50% mortality (LC_50_), conidial suspensions were adjusted to 1×10^5^, 1×10^6^, 1×10^7^, and 1×10^8^ conidia ml^−1^ using 0.03% (v/v) siloxane solution (Silwet L-77) and subjected to the same spray test as described above. Data on the percentage of live larvae was analyzed by a general linear model, followed by Tukey’s honestly significant difference, and median survival time and lethal concentration were determined with probit analysis using a SPSS ver. 17.0 (SPSS Inc., 2009) at the 0.05 (*α*) level.

## Supporting Information

Figure S1 **Flow chart of pAB-BbsVSP construction.** **(a)** Construction of pBluscript II KS(+)-egfp cassette. The 3.7 kb *egfp* expression cassette was cut from pABeG and inserted to pBluscript II KS(+). **(b)** Construction of pBluscript II KS(+)-Bbs-vsp cassette. The *Bbs-vsp* PCR product was inserted to the position of *egfp* in pBluscript II KS(+)-egfp cassette. **(c)** Construction of the binary plasmid pAB-Bbs-VSP. The *Bbs-vsp* expression cassette from pBluscript II KS(+)-Bbs-vsp cassette was inserted to the position of *egfp* expression cassette in pABeG.
(PDF)

Figure S2 **Degradation of fibrin in the wild type (Wt) and the BbsVSP-#181 transformant (#181) supernatant solutions 3 h of post-incubation at 37°C.** Supernatant was loaded at 200, 400, and 800 µl tube^−1^, where 100 µl fibrinogen (0.25%, in PBS) solution was clotted by 10 units of thrombin (1 unit/50 µl). Treated supernatant solution was completely removed and the amount of remaining fibrin was observed.
(PDF)

Figure S3 **Yellow spotted longicorn beetles injected with wild type (Wt) and BbsVSP-#181 transformant (#181) conidia at 40 µl (1×10^7^ conidia ml** ^−**1**^ **) per larva 2, 4 and 7 days after injection.** Phosphate buffered saline (PBS) solution was used as a base for all the treatments. In the BbsVSP-#181 treatment, small dark brown spots (arrows) were observed 2 days post-injection, followed by complete insect melanization without fungal outgrowth in 7 days, but the wild type-injected larvae turned pink as mycosis without dark spots and finally covered with fungal mycelial mass.
(PDF)

Table S1 **Comparison of virulence between wild type and BbsVSP-#181 against beet armyworm larvae in laboratory conditions.**
(PDF)

Table S2 **Primers used for four-round serial PCR to tale** ***Bi*** **serine protease domain (vsp) with** ***B. bassiana*** **signal (Bbs) fragment for chitinase, finally flanked with** ***Bam*** **HI at 3′-end.**
(PDF)
