# Supplementary figures and images for: Bumblebee Venom Serine Protease Increases Fungal Insecticidal Virulence by Inducing Insect Melanization
Source: PLoS One. 2013 Apr 23;8(4):e62555. doi: 10.1371/journal.pone.0062555 (PMC3633896; doi:10.1371/journal.pone.0062555)

Figure S1

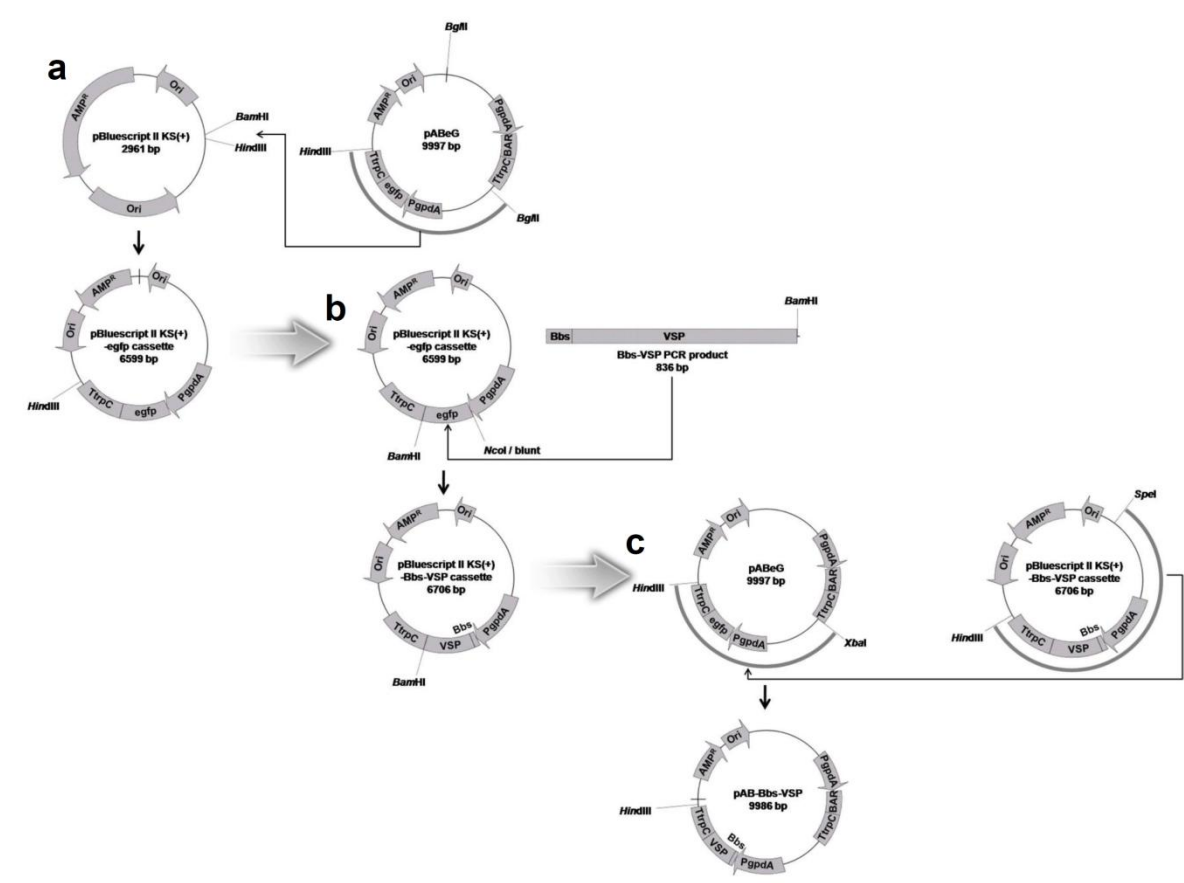

Supplement: Figure S1 — Flow chart of pAB-BbsVSP construction. (a) Construction of pBluscript II KS(+)-egfp cassette. The 3.7 kb egfp expression cassette was cut from pABeG and inserted to pBluscript II KS(+). (b) Construction of pBluscript II KS(+)-Bbs-vsp cassette. The Bbs-vsp PCR product was inserted to the position of egfp in pBluscript II KS(+)-egfp cassette. (c) Construction of the binary plasmid pAB-Bbs-VSP. The Bbs-vsp expression cassette from pBluscript II KS(+)-Bbs-vsp cassette was inserted to the position of egfp expression cassette in pABeG. (PDF) [file pone.0062555.s001.pdf]

**Figure S2**

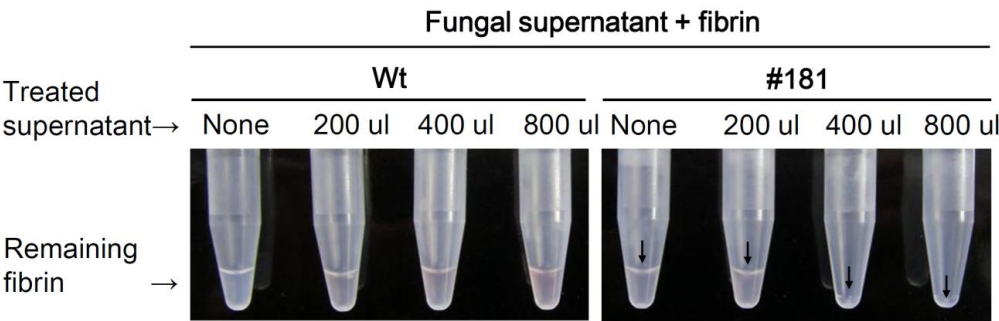

Supplement: Figure S2 — Degradation of fibrin in the wild type (Wt) and the BbsVSP-#181 transformant (#181) supernatant solutions 3 h of post-incubation at 37°C. Supernatant was loaded at 200, 400, and 800 µl tube−1, where 100 µl fibrinogen (0.25%, in PBS) solution was clotted by 10 units of thrombin (1 unit/50 µl). Treated supernatant solution was completely removed and the amount of remaining fibrin was observed. (PDF) [file pone.0062555.s002.pdf]

**Figure S3**

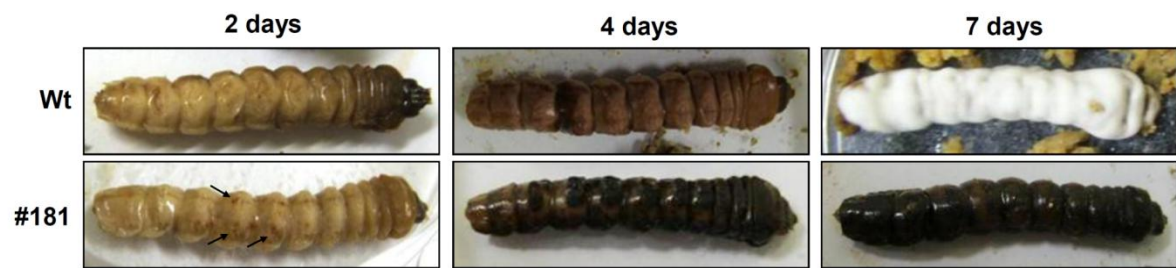

Supplement: Figure S3 — Yellow spotted longicorn beetles injected with wild type (Wt) and BbsVSP-#181 transformant (#181) conidia at 40 µl (1×10^7^ conidia ml −1 ) per larva 2, 4 and 7 days after injection. Phosphate buffered saline (PBS) solution was used as a base for all the treatments. In the BbsVSP-#181 treatment, small dark brown spots (arrows) were observed 2 days post-injection, followed by complete insect melanization without fungal outgrowth in 7 days, but the wild type-injected larvae turned pink as mycosis without dark spots and finally covered with fungal mycelial mass. (PDF) [file pone.0062555.s003.pdf]
