# Supplementary material for: Bumblebee Venom Serine Protease Increases Fungal Insecticidal Virulence by Inducing Insect Melanization
Source: PLoS One. 2013 Apr 23;8(4):e62555. doi: 10.1371/journal.pone.0062555 (PMC3633896; doi:10.1371/journal.pone.0062555)
Supplement: Table S1 — Comparison of virulence between wild type and BbsVSP-#181 against beet armyworm larvae in laboratory conditions. (PDF) [file pone.0062555.s004.pdf]

Table S1

	MST_{50} (days)* (95% confidence level)	LC_{50} ($\times 10^5$ conidia ml^{-1})** (95% confidence level)
Wild type	9.3 (6.8 – 11.8)	41.3 (24.2 – 58.4)
BbsVSP-#181	4.2 (3.4 – 5.0)	3.6 (2.8 – 4.4)

*MST_{50}: median survival time at 1×10^7 conidia ml^{-1} of spray treatment.

**LC_{50}: lethal concentration causing 50% mortality at 7 days after the spray treatments (1×10^5, 1×10^6, 1×10^7, and 1×10^8 conidia ml^{-1}).
