# Supplementary material for: Bumblebee Venom Serine Protease Increases Fungal Insecticidal Virulence by Inducing Insect Melanization
Source: PLoS One. 2013 Apr 23;8(4):e62555. doi: 10.1371/journal.pone.0062555 (PMC3633896; doi:10.1371/journal.pone.0062555)
Supplement: Table S2 — Primers used for four-round serial PCR to tale Bi serine protease domain (vsp) with B. bassiana signal (Bbs) fragment for chitinase, finally flanked with Bam HI at 3′-end. (PDF) [file pone.0062555.s005.pdf]

Table S2

Object	Primer	Sequence (5′-3′)
Bbs-vsp	1F	GCCTCGCCCTTGGCGCCGCGAGTGGTCGGTGGTAAGCCAGC
	1R	CCCGGGATCCTTATTGCATCGCTGGGAG
	2F	TTGGCTTCCACCATGGTCAGCGCCTCGCCCTTGGCGCCGC
	2R	CCCGGGATCCTTATTGCATC
	3F	AGCCTCGCGCTCCTTCCATTGTTGGCTTCCACCATGGTCAG
	3R	CCCGGGATCCTTATTGCATC
	4F	ATGGCTCCTTTTCTTCAAACCAGCCTCGCGCTCCTTCCAT
	4R	CCCGGGATCCTTATTGCATC

The underlined sequences indicate the regions of annealing parts to the templates. Initial denaturation at 95˚C for 3 min, 1 cycle with denaturation at 95˚C for 30 sec, annealing at 45˚C for 30 sec, polymerization at 74˚C for 1 min, 30 cycles with denaturation at 95˚C for 30 sec, annealing at 60˚C for 30 sec, at polymerization 74˚C for 1 min, and final extension at 74˚C for 5 min.
